# Alcohol Sensitivity as an Endophenotype of Alcohol Use Disorder: Exploring Its Translational Utility between Rodents and Humans

**DOI:** 10.3390/brainsci10100725

**Published:** 2020-10-13

**Authors:** Clarissa C. Parker, Ryan Lusk, Laura M. Saba

**Affiliations:** 1 Department of Psychology and Program in Neuroscience, Middlebury College, Middlebury, VT 05753, USA; 2 Department of Pharmaceutical Sciences, Skaggs School of Pharmacy and Pharmaceutical Sciences, University of Colorado Anschutz Medical Campus, Aurora, CO 80045, USA; ryan.lusk@cuanschutz.edu

**Keywords:** alcoholism, alcohol dependence, alcohol sensitivity, alcohol use disorder (AUD), animal models, cross species validation, endophenotype, genetics, genome-wide association studies (GWAS), rodents

## Abstract

Alcohol use disorder (AUD) is a complex, chronic, relapsing disorder with multiple interacting genetic and environmental influences. Numerous studies have verified the influence of genetics on AUD, yet the underlying biological pathways remain unknown. One strategy to interrogate complex diseases is the use of endophenotypes, which deconstruct current diagnostic categories into component traits that may be more amenable to genetic research. In this review, we explore how an endophenotype such as sensitivity to alcohol can be used in conjunction with rodent models to provide mechanistic insights into AUD. We evaluate three alcohol sensitivity endophenotypes (stimulation, intoxication, and aversion) for their translatability across human and rodent research by examining the underlying neurobiology and its relationship to consumption and AUD. We show examples in which results gleaned from rodents are successfully integrated with information from human studies to gain insight in the genetic underpinnings of AUD and AUD-related endophenotypes. Finally, we identify areas for future translational research that could greatly expand our knowledge of the biological and molecular aspects of the transition to AUD with the broad hope of finding better ways to treat this devastating disorder.

## 1. Introduction

Alcohol use disorder (AUD) is a chronic, relapsing disorder that causes significant harm to both the individual and to society [1]. The importance of genetic influences on AUD is well known [2,3,4]; yet identifying the underlying genetic mechanisms has proven difficult. In the past decade, human genome-wide association studies (GWAS) have revolutionized the field of psychiatric genetics and have successfully identified novel risk loci for numerous traits and diseases, including those with relevance to AUD [5,6,7,8,9,10,11,12,13]. However, human GWAS also face a number of challenges, including the need for extremely large sample sizes, the use of qualitative clinical diagnoses, inconsistent single nucleotide polymorphism (SNP)-trait associations, the discovery of SNPs with relatively small effects (i.e., missing heritability), and findings (either SNP associations or polygenic risk scores) that do not necessarily help elucidate the underlying biology [14,15,16,17].

One of the reasons that it has been difficult to identify the genetic underpinnings of AUD is due to the fact that AUD is a complex disease and its risk derives from multiple interacting genes, diverse environmental and lifestyle influences, gene-by-environment interactions, and differing combinations of psychiatric or other risk factors. Furthermore, the clinical definition of an AUD encompasses many symptoms, not all of which are required for a diagnosis. Thus, two individuals could receive the same diagnosis but not share any common symptoms. As Sanchez-Roige et al. [18] note, a diagnosis of AUD represents the end point of a series of stages that includes (1) initial sensitivity to the effects of alcohol, (2) the transition to hazardous alcohol use and the loss of control over alcohol-seeking and alcohol-taking, (3) the development of tolerance, and (4) relapse. Each of these domains is likely to be influenced by unique and perhaps non-overlapping networks of genes. Other aspects of AUD, such as activation of brain reward circuitry or personality traits, may be common across stages, across different drugs of abuse, or even across multiple psychiatric disorders [19]. In support of this, recent studies have utilized genomic structural equation modeling to suggest that common variance shared across psychiatric disorders, personality traits, and nonclinical behaviors appears to be related to risk for externalizing problems [20,21,22,23]. Furthermore, it is possible that the genetic risk specific to AUD explains a relatively smaller proportion of the variance than what is shared.

An alternative strategy to interrogating complex diseases that is gaining traction in large-scale AUD GWAS involves using endophenotypes (see [9,24]). Endophenotypes attempt to deconstruct current diagnostic categories into quantitative underlying traits that are more amenable to genetic research [25]. As a result, the causal genes or gene networks should be easier to identify, because they will have a larger effect on the endophenotype than on the psychiatric disorder. To maximize their translational utility, Gould and Gottesman [26] proposed that endophenotypes be (1) associated with illness in the population, (2) heritable, (3) state-independent, (4) cosegregate with the disorder within families, and (5) present at higher rates within affected families as compared to the general population.

**Figure 1:**
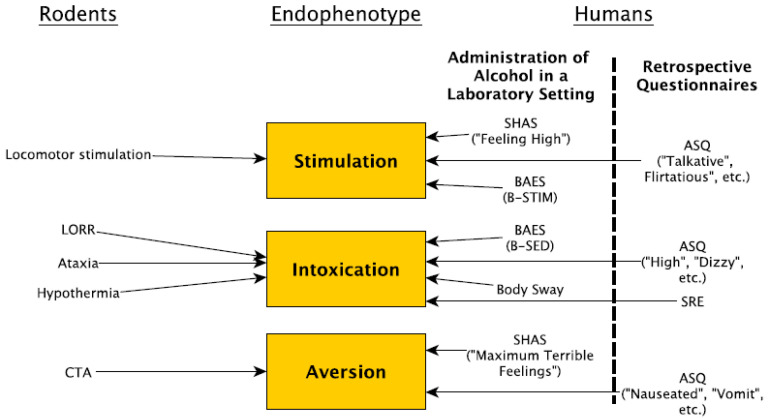
Tests used to evaluate the endophenotypes stimulation, intoxication, and aversion in humans and rodents. For humans, the Subjective High Assessment Scale (SHAS), Alcohol Sensitivity Questionnaire (ASQ), and Biphasic Alcohol Effects Scale (BAES) evaluate more than one endophenotype. In addition, body sway is used to measure intoxication in humans. For rodents, locomotor stimulation and conditioned taste aversion (CTA) assess stimulation and aversion, respectively, and multiple tests are used to measure intoxication including loss of righting response (LORR), ataxia, and hypothermia.

In this review, we will explore how one endophenotype—sensitivity to alcohol (also referred to as subjective response (SR) in humans)—can be used in conjunction with rodent models to provide mechanistic insights into AUD. While the entire spectrum of any human psychiatric disorder cannot be fully recapitulated in a single rodent model, there is substantial behavioral, genetic, and neuroanatomical conservation between humans, mice, and rats. Furthermore, when broken down into individual components, many aspects of an AUD can readily be modeled in rodents. Here, we will evaluate how both alcohol sensitivity and rodent models can be integrated into this framework (Figure 1). At a time when some GWAS researchers are questioning the value of model organisms, we argue that translational rodent research can provide a powerful strategy for understanding the genetic and biological underpinnings of AUD and are complementary to human studies.

## 2. Alcohol Sensitivity in Humans

There is considerable variation across individuals in their sensitivity to the intoxicating effects of alcohol [27]. This variability has been linked with the later development of AUD [28,29,30,31,32] and is known to be at least partly genetically mediated [33,34,35]. The subjective response is characterized by an initial stimulant phase during the ascending limb of the blood alcohol concentration (BAC) curve, followed by the sedative phase during the descending limb of the BAC curve [36]. Thus, sensitivity represents the interplay between both pleasurable and aversive effects of alcohol, which over the course of repeated alcohol exposures, may function as a determinant of future alcohol intake and AUD risk. Decreased sensitivity to the unpleasant subjective effects of intoxication (such as ataxia and sedation) may contribute to increased risk for AUD, as individuals who experience fewer aversive intoxicating effects of alcohol are more likely to escalate drinking behavior [37,38]. This is commonly referred to as “low level of response” (low LR) and is a significant predictor of later alcohol consumption [30,39], likely because a lack of sensitivity allows individuals to consume large quantities of alcohol without any negative effects. In addition, increased sensitivity to the positive subjective effects (such as enhanced stimulation and reward) has been associated with a greater probability of developing an AUD [40]. While low LR and enhanced stimulation to alcohol have been observed in the same individuals, the factors that underlie them may be at least partly distinct [28,41,42,43,44]. Therefore, according to the Differentiator Model [28], they may each represent unique endophenotypes that fall under the broad category of subjective response and differentially predict risk for AUD. Variation in alcohol sensitivity may not only predict future consumption; it may also predict the success of treatment programs. For example, college-based prevention programs that target LR are effective in individuals with low, but not high, LR [45,46]. Thus, identifying genetic variation underlying alcohol sensitivity could not only help identify individuals at higher risk of AUD, but could also aid in developing targeted interventions to reduce alcohol consumption.

In humans, alcohol sensitivity was traditionally measured in a laboratory setting by administering an alcohol challenge (either orally or intravenously) and assessing measures of body sway and self-reported subjective high (as measured by the Subjective High Assessment Scale, or SHAS) over several hours. The SHAS consists of both positive and negative mood-related descriptors but is most sensitive to the unpleasant effects of alcohol [47]. Other laboratories utilize the Biphasic Alcohol Effects Scale (BAES). The BAES is a 24-item self-report questionnaire that examines seven items, each regarding sedation and stimulation during alcohol challenges. However, laboratory-based alcohol challenges are expensive and time-consuming, making it difficult for researchers to obtain a sufficiently large sample size. As a result, they have not been widely utilized in large scale human GWAS, even though they have been of great interest to the alcohol research community for many years [48]. Furthermore, most alcohol challenge studies recruit participants that are at least 21 years of age and whose responses may be influenced by prior experience [49]. Consequently, laboratory-based alcohol challenges are not able to test individuals during vulnerable developmental periods and may not be obtaining measures of LR at first exposure to alcohol. To partly overcome these limitations, many researchers measure alcohol sensitivity by the Alcohol Sensitivity Questionnaire (ASQ) or by the Self-Report of the Effects (SRE) of Alcohol Questionnaire [39,50,51,52,53]. The ASQ consists of 15 questions designed to separately measure stimulation and sedation. It asks participants to retrospectively report if they ever experienced a given effect, and if so, the minimum or maximum number of drinks associated with the item. In the SRE, subjects retrospectively estimate the number of drinks usually required to experience various effects (feel an effect; feel dizzy/slurred speech; stumbling/uncoordinated walking; pass out/fall asleep) of alcohol at three different timepoints (the individual’s first five times of drinking, the period in which the individual engaged in their heaviest drinking, and the individual’s most recent three months of consumption). While the SRE encompasses intoxication rather than stimulation, it is moderately (r = 0.3–0.6) correlated with LR measured in a laboratory, and has high levels of test–retest reliability (r = 0.66–0.82) and consistent predictive validity [54,55,56,57]. Importantly, Johnson et al. [58] showed that polygenic risk scores (PRS) obtained from a GWAS on alcohol consumption did not explain a significant amount of variance in measures of the SRE, suggesting that consumption and LR may have unique genetic underpinnings in humans. This is somewhat surprising, given previous research suggesting that low LR is predictive of future problems with alcohol primarily via heavy consumption [30,38,47,59,60]. One explanation for the weak association between PRS for alcohol consumption and SRE could be due to the low power of the discovery sample (~115 k subjects), as well as differences in the genetic architecture between populations. In support of this explanation is the finding that the consumption PRS used by Johnson et al. [58] did not strongly predict alcohol consumption (~1.5%) across populations. Recently, Lai et al. [61] conducted a GWAS and meta-analysis for SRE scores. They identified a number of significant associations and found that SRE scores were moderately heritable (*h*^2^ = 21–31%) and genetically correlated (*r* = 0.35–0.76) with alcohol dependence (AD) and DSM-IV AUD symptom count [61]. Their findings underscore the value of using an endophenotype such as SR for genetic discovery. However, it is important to note that PRS for SRE scores only predicted a small proportion (0.47–2.48%) of the variance in AD and DSM-IV AUD symptom count when applied to independent datasets, suggesting that there may be only modest overlap between genetic variants associated with SRE and those linked to AUD diagnosis. Similar results have been found for the endophenotype of alcohol consumption, in which genetic differences and opposite genetic correlations were reported between alcohol consumption measures and AUD [61]. Thus, although SR may be a significant risk factor for the development of AUD, the latter is a polygenic disorder that results from multiple cycles of binging and intoxication, the development of tolerance, and relapse. Therefore, many additional gene networks are likely to be recruited/disrupted for an individual with altered alcohol sensitivity to ultimately develop an AUD. Still, retrospective questionnaires like the SRE and the ASQ are particularly amenable to large-scale GWAS and may provide useful, unique endophenotypes for AUD that are distinct from consumption. Future research that systematically compares the relationships between alcohol sensitivity (both LR and stimulation), other alcohol-related endophenotypes, and AUD diagnosis in well-powered studies can enhance our understanding of the origins and trajectories of AUDs and lead to targeted prevention and treatment strategies.

## 3. Advantages of Rodent Models

Rodent models have a number of advantages for identifying the biological mechanisms underlying AUD endophenotypes. First, environmental factors can be held constant, systematically manipulated, or statistically accounted for in order to examine interactions between genotype and environment. Reducing the error variance (such as that due to the effects of environmental variation) on clinically salient traits in turn increases statistical power, thus permitting a greater proportion of trait variance to be attributed to genetics. This is hypothesized to be one reason why many rodent GWAS regularly report effect sizes that are significantly larger than those in human GWAS [62,63,64,65]; but see [66]. Second, researchers can cross strains with measurable phenotypic differences and quickly generate large populations of offspring from a limited number of founder genotypes [67]. In addition, both the mouse and rat genomes have been sequenced and annotated [68,69,70,71,72]. Using orthology information from the Genome Alliance, 95% of human protein-coding genes have evidence of an ortholog in mouse and 94% in rat [73,74]. This allows rapid translation between mice, rats, and humans. Furthermore, putative medications can be rapidly screened in rodent models at different stages in the addiction cycle in order to determine optimal therapeutic strategies relative to stage in the addiction cycle [75]. Another significant advantage is that brain tissue can be collected under idealized laboratory conditions, at specific treatment or developmental time-points, and following invasive procedures. This neuronal tissue can then be used to measure gene expression and map expression quantitative trait loci (eQTLs), which is particularly relevant given that regulatory polymorphisms appear to underlie the vast majority of causal loci in human GWAS [76,77]. In contrast, human postmortem brain tissue (when available) is most commonly representative of the cumulative effects of end-stage AUD. Furthermore, comparing equivalent brain tissue samples from human subjects is difficult given the vastly different life histories and substance exposures in individuals with AUD. This heterogeneity makes obtaining perfectly matched control tissue virtually impossible, which in turn may confound the analysis of gene expression.

## 4. Genetic Approaches in Rodent Models of AUDs

Another significant advantage of rodent models for studying AUD is the ability to utilize methods for both forward and reverse genetics approaches. Forward and reverse genetics approaches represent opposite but complementary strategies for studying the genetic basis of AUD. In a forward genetics approach, the study begins with measuring a phenotype of interest and asks which genes influence a phenotype in a particular population [76]. Reverse genetics approaches allow researchers to examine the function of a gene by quantifying the phenotypic effects that result from a targeted manipulation of that gene. Both methods are useful for integrating results from human and rodent genetic studies.

### 4.1. Forward Genetics

Forward genetic strategies attempt to identify genetic variants that underlie variability in a trait of interest. Their major advantage relies on their unbiased nature; no assumptions are made regarding the molecular basis of the phenotype under investigation [40]. Two common forward genetic approaches include (1) selective breeding (summarized in [78]), and (2) quantitative trait locus (QTL) analysis [79].

In selective breeding paradigms, animals from a genetically diverse population are phenotyped for a trait of interest and bred on the basis of their level of response. In bidirectional breeding strategies, the resulting progeny then represent high and low lines for that trait. The larger the genetic influence on the trait is, the greater the spread between the diverging lines. Once the lines are highly divergent for the selection phenotype, researchers can not only examine the genetic differences that exist between lines, but also determine whether the selected alleles influence other potentially related phenotypes. The alcohol research community had taken advantage of this approach for many years [78]; and selective breeding for a wide range of ethanol sensitivity-related behaviors has been conducted in mice and rats [80,81,82,83,84,85,86,87,88,89]. While this approach has been used for QTL mapping in the past, it generally does not provide sufficient resolution for gene identification. Rather, it is most useful for determining the heritability of a trait, for identifying correlated responses to selection, and for identifying gene coexpression networks that differ between high and low selected lines [78].

QTL mapping is another forward genetic approach for identifying alleles that contribute to variation in phenotypes. To perform QTL mapping, a large number of subjects are phenotyped and genotyped across the entire genome to identify variants (usually SNPs) that are associated with the trait. The implicated SNP is not necessarily the causal polymorphism; rather, it “tags” a region (locus) that contains the causal variant. To be successful, this approach not only requires phenotypic and genomic variation within the population being studied, but also sufficient amounts of genetic recombination to ensure that the tagged SNP is physically close to the causal SNP. There are several types of highly recombinant populations that can be used for rodent QTL studies that have gained traction in recent years due to advances in genotyping technology and statistical techniques [90]. They include heterogeneous stocks (such as the heterogeneous stock (HS) rat and mouse populations), hybrid diversity panels (such as the Hybrid Rat Diversity Panel (HRDP) and Hybrid Mouse Diversity Panel (HMDP)), recombinant inbred (RI) strains (such as the BXD, LXS, and CC panels), and commercially available outbred stocks (such as CFW mice and Sprague Dawley rats) [62,91,92,93,94,95,96,97,98,99,100,101,102]. Heterogeneous stocks and commercially available outbred populations are generally created by semi-random breeding strategies that maximize genetic diversity while maintaining favorable minor allele frequencies (e.g., >10%). Furthermore, because these populations have been allowed to naturally recombine over many generations, the haplotype blocks in these populations can be quite small due to the breakdown of linkage disequilibrium. In turn, this allows for fine-resolution mapping. RI panels are created by crossing two or more inbred strains to produce the F_1_ generation. This is followed by a minimum of 20 consecutive generations of brother–sister matings to obtain the different RI strains within a panel. Each RI strain within a given RI panel is homozygous at each locus throughout their genome, and each pair of strains within a RI panel has a relationship similar to dizygotic twins (i.e., they share 50% of their genetics). Hybrid diversity panels are collections of well characterized classic inbred strains and RI strains that can be used to map QTLs and identify genes associated with complex traits.

Each mapping population possesses unique strengths and weaknesses. For example, heterogeneous stocks and outbred populations provide almost limitless power and precision. However, one disadvantage is that every animal must be genotyped and phenotyped, which can be cost prohibitive. In addition, once a given animal has been sacrificed or exposed to the independent variable (e.g., alcohol, stress), their unique genotype cannot be reproduced for further study. Therefore, all measures must be obtained from the same animals. Hybrid diversity panels and RI strains can overcome these issues. Because they are inbred, multiple biological replicates of a given background can be examined to segregate non-genetic variance in the trait from genetic variation of that trait between strains (i.e., across different genetic backgrounds). Furthermore, the genetic composition of hybrid diversity panels and RI strains is retained over generations. Therefore, any data collected (e.g., genome sequence, RNA expression levels, phenotypes) can be compared over time and across laboratories for more integrative analyses. However, because they are derived from just two parental strains, traditional two-parent RI panels only segregate a small proportion of all known polymorphisms. Therefore, many researchers have turned to multi-parental genetic reference panels in order to increase the genetic diversity of their mapping population [103]. We recommend that researchers first evaluate how the unique characteristics of each mapping population can best address their question of interest before embarking on a forward genetics study [104]. It may be the case that scientists should use more than one type of population in a discovery/validation approach. Other genetic populations that will not be discussed in this review but that can also be leveraged in a forward genetics approaches include genome-wide *N*-ethyl-*N*-nitrosourea (ENU) mutagenesis panels [105], reduced complexity crosses [106], and advanced intercross lines [107].

Both RI panels and heterogeneous stocks [94,108,109,110,111,112,113,114,115,116,117,118,119] have been extensively studied for alcohol-related phenotypes. Furthermore all of these populations have been successfully used to map genes associated with psychiatric traits [63,91,100,101,120,121,122,123,124] including addiction-related phenotypes [125]. It should be noted that the same advances in genotyping technologies that made human GWAS possible have also revolutionized rodent genetics. It is now inexpensive and fairly straightforward to perform GWAS using rodents. Therefore, using forward genetics approaches in highly recombinant rodent populations represents an unrealized opportunity to identify genes associated with alcohol-related traits. These genes and their networks can then be studied in human populations to determine whether they contribute to variation in corresponding endophenotypes.

### 4.2. Reverse Genetics

In contrast to forward genetics, reverse genetics begins by observing the impact of gene perturbation(s) on a phenotypic outcome hypothesized to be influenced by the gene. Reverse genetic approaches include gene ablation (knockouts), alterations/replacements of genes (knockins), insertion of extra copies of genes (transgenics), or the use of short interfering RNA segments (siRNA knockdowns). These techniques require a major disruption to a single gene, and may poorly model the often subtle and widely distributed genetic variations that result in complex, polygenic phenotypes [126]. This limitation has been overcome to some degree by the use of conditional and inducible systems that permit tissue- or developmentally-specific changes in gene expression by exogenous triggers [127,128,129]. The reverse genetic approach can yield rich understanding into the role of particular genes and provide mechanistic explanations of genetic hypotheses. Indeed, high-throughput reverse genetic approaches are a major tool for characterizing the functional consequences of genes. However, unlike forward genetics, reverse genetics is inherently biased in that genetic mutations are chosen prior to phenotype analysis. In addition, since most genes interact with other genes that are themselves polymorphic, mutating one gene may result in different phenotypic outcomes based on different genetic backgrounds at other loci [130]. Therefore, in order to optimize the translational value of reverse genetic approaches in rodents, any putative genotype–phenotype relationships should be validated in multiple genetic backgrounds. Traditionally, mice were the most commonly used model organism for most reverse genetic approaches, whereas rats lacked tools for genome modification. However, nuclease-mediated targeting by clustered regularly interspaced short palindromic repeats (CRISPR/Cas), transcription activator-like effector nucleases (TALENs), and zinc-finger nucleases (ZFNs) have been developed to quickly and efficiently modify genes across different cell types and model organisms, including rats [131,132,133,134,135]. In fact, there are now a number of research groups around the world that are taking advantage of these new genome-editing tools to generate rat knockouts that can provide insight into human disorders [136]. Given the development of genome-editing technologies that can now be used in either mice or rats, AUD researchers should carefully consider the physiological, neuroanatomical, and behavioral differences between the two species when selecting a model system for a translational study.

## 5. Alcohol Sensitivity in Rodents

Alcohol sensitivity can be measured in rodents in a number of ways and provides a promising point of entry for human/rodent translational genetic studies. However, in order for the knowledge gleaned from rodent models to be applicable to the human condition, it is necessary to first understand which measures of sensitivity might be translatable across species and to identify areas where further research is needed. In the paragraphs below, we evaluate three alcohol sensitivity phenotypes that are commonly compared between humans and rodents: (1) stimulation, (2) intoxication, and (3) aversion. Similar to what has recently been reported in human GWAS [58], data supporting a relationship between alcohol sensitivity and consumption in rodents is mixed. For each phenotype, we describe its underlying neurobiology, evaluate its strengths and weaknesses in modeling SR in humans, discuss its relationship to consumption, and provide examples in which the phenotype has been successfully used in rodents to provide insight into the human condition.

### 5.1. Ethanol-Induced Locomotor Stimulation

One commonly used behavioral assay of sensitivity is locomotor stimulation in response to ethanol. Here, rodents are injected with a low dose of ethanol, and their activity is recorded over time. Many drugs of abuse, including alcohol [137], increase locomotor activity in rodents. Sensitivity to ethanol’s locomotor activating effects is partially mediated by dopamine release in mesolimbic brain regions [138] and has been posited to be reflective of the stimulating/rewarding effects of ethanol [27,139,140,141,142]. This endophenotype has been useful for human/rodent translational genetic studies, but it is not without limitations. One potential caveat is that while alcohol increases subjective reports (e.g., pleasure, stimulation, euphoria) of reward, it does not specifically increase locomotor activity at recreational doses in humans. However, brain imaging studies in humans have demonstrated that ethanol-induced increases in dopamine in the nucleus accumbens and ventral striatum are well correlated with these self-reported subjective responses [143,144,145]. Thus, while it is lacking in face validity, locomotor activity in rodents can successfully be used to estimate the degree of dopaminergic activation in the mesolimbic pathway, which likely reflects the same process that makes alcohol rewarding in humans. Rodent QTL mapping studies have identified genomic regions associated with differential sensitivity to the locomotor activating effects of ethanol [146,147,148,149,150,151,152,153]. In addition, there are selectively bred mouse lines (FAST and SLOW [80,86]) that differ in their sensitivity to the locomotor activating effects of ethanol, and rat lines that have been selectively bred for ethanol-induced depression of locomotor activity [82,88,89]. Multiple groups have used these selectively bred lines to study the pleiotropic effects of genes that mediate other responses to ethanol, such as consumption. For example, Risinger et al. [154] reported that FAST mice have increased consumption and preference for ethanol solutions in home-cage, two-bottle choice procedures as compared to SLOW mice, but others have observed no significant differences between FAST and SLOW mice in the amount of work produced to obtain ethanol or in the amount of ethanol consumed in self-administration tasks [155]. Others have approached this question from the opposite direction; they have bred rodent lines for differences in ethanol preference/consumption and then tested them for the stimulant effects of ethanol. In some instances, ethanol induces greater amounts of locomotor activation in rodents that have been selectively bred for high preference/consumption [156,157,158,159,160], but in other cases it does not [153,161]. While these incongruent results may simply be due to methodological differences between various consumption assays, to differences in developmental time points at which animals were tested, or to the influence of prior ethanol exposure, additional research is needed to clarify the relationship between ethanol-induced locomotor activation and subsequent ethanol consumption.

A large body of evidence from rodent studies has identified the large conductance voltage and calcium-sensitive potassium (BK) channel as being involved in moderating the behavioral effects of ethanol, including ethanol-induced locomotor activation. *Kcnma1* encodes the α subunit of the BK channel. In addition, a β subunit (β4) interacts with the α subunit of the BK channel to alter its conformational and pharmacological properties; it is responsive to ethanol and is encoded in mammals by the *Kcnmb4* gene [162,163,164]. Kerns et al. [165] performed a transcriptome-wide study that identified a BK channel subunit associated with ethanol-induced activation. In the study, researchers examined gene expression in multiple brain regions (nucleus accumbens, prefrontal cortex, and ventral tegmental area) following acute ethanol or saline injection in two mouse strains (C57BL/6J and DBA/2J); they showed large differences in ethanol-induced locomotor activation and alcohol consumption [166]. Specifically, DBA/2J mice showed significant ethanol-induced locomotor activation and decreased ethanol consumption compared to C57BL/6J mice. *Kncma1* was one of only 307 genes whose RNA expression levels were altered in response to ethanol in either strain in any of the brain regions. In particular, the expression of *Kcnma1* was upregulated in the nucleus accumbens of DBA/2J mice but not C57BL/6J mice. Gene expression differences were then examined in regions of the mesocorticolimbic reward circuit (prefrontal cortex, nucleus accumbens, and ventral midbrain) of the BXD RI panel [167] before and after ethanol administration. Not only did ethanol influence *Kcnma1* gene expression in multiple brain regions, but additional bioinformatic analyses of gene network connectivity identified *Kcnma1* as a hub gene. Given that hub genes represent the most highly connected genes in a coexpression network, these results suggest that it may be a major regulator of the transcriptional response to ethanol. Importantly, *Kcnma1* was differentially expressed across the RI strains, thus implying that there are genetic differences in the mechanism of regulation. There is also evidence from several human genetic studies implicating *KCNMA1* in LR and alcohol dependence. For example, Schuckit et al. [59] performed a linkage analysis for LR in 238 sibling pairs and found that the *KCNMA1* gene locus was associated with ratings of subjective high as measured by SHAS. While multiple SNPs in the *KCNMA1* gene showed significant associations with the SHAS measure, they failed to reach significance after correction for multiple testing, likely due to the small sample size. However, subsequent larger GWAS have also identified suggestive and significant associations with *KCNMA1* and alcohol dependence [168,169,170], thus providing additional support for this association. To explore the underlying mechanism, researchers in [171] measured ethanol-induced locomotor activation and consumption in mice with a null mutation in the *Kcnmb4* gene (KO). Following acute ethanol administration, *Kcnmb4* KO mice developed rapid tolerance to ethanol’s locomotor activating effects and consumed more ethanol in restricted access self-administration paradigms compared to wildtypes [171,172]. Evidence showing the molecular mechanism by which this occurs comes from single channel recordings in HEK-293 cells and in medium spiny neurons in the mouse ventral striatum, which together indicate that in the absence of β4, cells develop acute tolerance to ethanol potentiation of activity [171]. Thus, a knockout mouse model of ethanol-induced locomotor activity helped to clarify the role of the BK channel, which in turn provides mechanistic insight about the importance of α and β subunits in a cell type- and brain region-specific manner—a result that would have been difficult if not impossible to obtain using human subjects alone. Together, these bioinformatic, forward, and reverse genetics approaches suggest that (1) BK channels may exert major influence over the ethanol response of gene networks, and (2) allelic variation in the genes encoding the subunits of these BK channels can influence both LR and subsequent risk for developing AUD in humans and represents a promising target for intervention.

### 5.2. Ethanol-Induced Intoxication (Ataxia, LORR, Hypothermia)

Another approach to examine SR in rodents is to measure their sensitivity to the intoxicating effects of ethanol. There are a number of somewhat disparate assays that purport to quantify response to the intoxicating effects of ethanol, and they include assays for ataxia, loss of the righting response (LORR), and hypothermia [173]. Furthermore, it is important to note that despite the fact that ataxia, LORR, and hypothermia tests are all considered to be indicators of “intoxication”, they rely on distinct neural circuits and show only modest genetic correlations—both within different assays that purport to measure the same phenotype (e.g., rotarod vs. grid ataxia) and across intoxication phenotypes (e.g., ataxia vs. LORR; see [174]). Thus, there is no single behavioral assay that best measures “intoxication” in the broad sense. As a result, a test battery may be required to more fully map the neurogenetic features of response to the intoxicating effects of ethanol.

#### 5.2.1. Ataxia

Rodent behavioral assays that measure ataxia generally assess sensitivity to the motor incoordinating effects of ethanol, which are widely regarded as unpleasant in humans [175]. Ataxia paradigms attempt to model the body sway or ataxia that a person exhibits following consumption of alcohol in an alcohol challenge or as reported in the SRE and ASQ. However, in humans, motor impairment peaks while stimulation levels are at their highest point in the rising limb of the BAC curve, and motor coordination recovers as sedative effects are most pronounced [176,177]. Adding to this complexity is research indicating that ethanol-induced motor incoordination is enhanced in genetically at-risk individuals if they are assessed on the rising limb of the BAC curve, but attenuated if they are evaluated on the descending limb of the BAC curve [178]. In addition, the neurocircuitry underlying the ataxic effects of alcohol in humans has not been fully elucidated, although it is hypothesized that decreased functioning in the cerebellum and frontal lobe is responsible [179,180,181]. A better temporal understanding of the brain mechanisms associated with ethanol-induced ataxia could help clarify whether motor incoordination reflects stimulation or sedation.

A number of studies have demonstrated that ethanol-induced ataxia is genetically mediated in rodents [182,183,184,185]. These ataxia tests typically involve administering low to moderate doses of ethanol and then measuring variables such as latency to fall from a screen (screen test), number of foot slips on a wire mesh floor (grid test) or balance beam (balance beam test), or latency to fall from a fixed or rotating dowel (dowel test, rotarod test; see [174]). While ataxia tasks presumably measure balance, some tasks (such as the rotarod) also recruit neurocircuitry involved in learning and motivation [186]. Given that so little is understood about the neurocircuitry responsible for body sway, it is not known which rodent ataxia assay best models indices of body sway in humans. Thus, ataxia represents a phenotype that should be systematically explored in human research so that appropriate ataxia assays in rodents can be identified and/or developed. Lastly, because there is uncertainty regarding the stimulant vs. sedative effects of ethanol on motor impairment, researchers should consider measuring blood ethanol concentration (BEC) levels across multiple time-points post-ethanol administration. Mapping ataxia along the rising and descending limbs of the BEC curve will allow for the assessment of individual differences in ethanol-induced ataxia and achieve better concordance with human studies.

The relationship between sensitivity to ethanol-induced ataxia and ethanol consumption in rodents is complex. Selectively bred high alcohol-preferring (HAP) mice were significantly more sensitive to the ataxic effects of ethanol on the static dowel test compared to low alcohol-preferring (LAP) mice [187]. In addition, BEC was measured immediately after loss of balance (on the ascending limb of the BEC curve) and at recovery (during the descending limb of the BEC curve). Not only did HAP mice fall from the dowel significantly earlier than LAP mice, they did so at lower BECs, suggesting that they were more sensitive to the early effects of ethanol [187]. However, work from the same lab has shown that mice (HDID-1) selectively bred for high BECs during drinking in the dark paradigm displayed reduced sensitivity to ethanol-induced ataxia and recovered at significantly higher BECs on the same static dowel test relative to HS/Npt mice [188]. It is possible that the discrepancy between these selectively bred mouse lines may simply be due to the different progenitor stocks from which they were derived, resulting in differing combinations and frequencies of segregating alleles. An alternative explanation is that selectively breeding for binge ethanol consumption produces unique responses to ethanol-induced ataxia as compared to selectively breeding for other forms of excessive ethanol consumption. As the authors note, both lines of mice are valuable models for the human condition. Blunted sensitivity to the adverse effects of ethanol (like that observed in HDID-1 mice) may allow at-risk individuals to consume large amounts of alcohol in a short period of time, whereas predisposition for sustained excessive alcohol consumption (as seen in HAP mice) may result in an enhanced capacity to binge drink over extended periods of time [187,188]. Thus, these selectively bred mouse lines provide the opportunity to systematically dissect the genetic mechanisms underlying different paths to excessive alcohol consumption.

Recently, ethanol-induced ataxia was examined in genetically modified mice to demonstrate that the inhibitory site for ethanol in a lesser studied GABA_A_ receptor subunit promotes tolerance to ethanol-induced motor incoordination. It has long been recognized that many effects of ethanol in vivo are mediated by enhancing the functions of GABA_A_ receptors, which are primarily composed of two α, two β, and one γ subunit [189,190,191,192]. However, the responses of GABA_A_ ρ receptor subunits are inhibited by ethanol [193], but they have not been well characterized in the brain. In the last decade, AUD genetics researchers have shifted their focus to the GABA_A_ ρ receptor when a family-based association analysis identified two genes that encode ρ1 and ρ2 receptor subunits (*GABRR1* and *GABRR2*) were associated with alcohol dependence in humans [194]. Others have exploited bioinformatics resources to identify correlations between ρ1 expression levels in the nucleus accumbens and ethanol-related behaviors in BXD RI mice (http://genenetwork.org; [146,195,196,197]). In order to explore the mechanism by which ethanol exerts its effects on ρ receptor subunits and influences risk for AUD, Blednov et al. [197] measured several ethanol-related behaviors in wildtype mice after administration of the ρ1 antagonist, (*S*)-4-amino-cyclopent-1-enyl butylphosphinic acid, and in ρ1 KO mice. Both the ρ1 KO mice and the pharmacologically treated wildtype mice showed faster recovery from ethanol-induced motor incoordination on the fixed speed rotarod as compared to controls [197]. In addition, ρ1 KO mice displayed reduced ethanol consumption/preference and increased duration of LORR. Based on this evidence, researchers then generated ρ1 knockin (KI) mice using CRISPR/Cas9 to elucidate the role of the inhibitory actions of ethanol on the ρ1 receptor [195]. They found that inducing a mutation in the ρ1 subunit of KI mice changed the inhibitory effects of ethanol into potentiated GABA responses while still maintaining normal channel function and normal levels of ρ1 and ρ2 receptors in the cerebellum. Similar to the ρ1 KO mice, ρ1 KI mice displayed a faster recovery from and increased tolerance to ethanol-induced motor incoordination. However, the ρ1 KI mice did not differ from wildtypes in ethanol consumption, conditioned taste aversion, or LORR duration [195]. Because these ethanol-related traits were altered in the ρ1 KO but not the ρ1 KI mice, Blednov et al. [195] concluded that the ρ1 subunit is important for these behaviors due to its intrinsic role in neuronal inhibition, rather than ethanol’s inhibitory actions on the receptor itself. Thus, comparing the results from ρ1 KO and ρ1 KI mice allowed researchers to elegantly disentangle the influence of loss of receptor function from ethanol’s inhibition of function and provided a mechanistic explanation for the GWAS finding of a genetic association of ρ1 and ρ2 subunits with AUD in humans.

#### 5.2.2. LORR

Duration of LORR is also used to measure intoxication and can be used to quantify sensitivity to the sedative effects of ethanol. Mice and rats have a righting reflex that corrects the orientation of their body when taken out of its normal, upright position. Given that ethanol is a central nervous system depressant that produces sedative–hypnotic effects on behavior, LORR can be used to estimate hypnotic sensitivity to ethanol in rodents and has been shown to be genetically influenced [83,85,108,182,183,185,198,199,200,201,202,203,204,205,206]. The LORR paradigm entails injecting animals with an anesthetic dose of ethanol, placing them on their backs in a V-shaped trough, and recording the amount of time it takes to regain the righting reflex as well as BEC at recovery [85]. Researchers have not fully identified the mechanisms that mediate alcohol-induced sedation in humans, but there is some evidence that alcohol causes a general decrease in activity throughout the cerebral cortex [141,207]. At the molecular level, the sedative effects of ethanol are associated with hypoactive NMDA receptors and hyperactive GABA receptors [208]. The LORR assay has poor face validity for the body sway indices that are collected in laboratory alcohol challenges. One reason for this is because laboratory alcohol challenges administer low to moderate doses of alcohol that are not sufficient to induce hypnotic effects. However, outside of the laboratory, it is clear that many people (including those with AUDs) do drink to the point of experiencing hypnotic effects [209]. Furthermore, retrospective studies using the SRE reported a highly significant negative correlation between subjective feelings of low dose intoxication and the number of drinks required to pass out early in subjects’ drinking histories [39]. Therefore, LORR appears to more closely align with individuals’ ratings of intoxication or other subjective negative effects (e.g., feeling nauseous or sick) as well as other measures collected by the SRE and ASQ (e.g., number of drinks consumed to induce passing out/falling asleep). These findings suggest that sensitivity to the sedative–hypnotic effects of high doses of alcohol can predict AUD risk in humans.

Multiple studies have examined the genetic relationship between LORR sensitivity and ethanol consumption in rodents [116,188,203]. For example, selectively bred mice that consume large amounts of ethanol (HDID-1 mice [78]) are significantly less sensitive than controls to ethanol’s sedative–hypnotic effects [188]. However, work in inbred long and short sleep (ILS and ISS, respectively) and LXS RI mice suggests that this relationship may be dependent on prior exposure to ethanol. ILS and ISS mice were selectively bred for differences in LORR duration [85]. Subsequent work [210] has shown that a large portion of the difference in LORR sensitivity between ILS and ISS mice is mediated by the development of tolerance within a single exposure (known as acute functional tolerance, or AFT). In the LORR paradigm, AFT is measured by comparing BEC at the initiation of LORR to BEC at LORR recovery. Radcliffe et al. [210] also assessed rapid tolerance (tolerance that is evident up to at least 24 h after a single administration of ethanol) by comparing LORR duration between groups of mice that received a pretreatment of either saline or ethanol 24 h prior to the LORR test. They observed a pronounced genetic effect on rapid tolerance, in that LORR duration was significantly reduced in ILS but not ISS mice 24 h following a single dose of ethanol [210]. Next, they explored the genetic relationship between AFT, rapid tolerance, and drinking behavior in the LXS RI panel [116]. Duration of LORR was not significantly correlated with ethanol consumption. However, ethanol consumption was significantly correlated (*r* = 0.64) with AFT, but only in the ethanol-pretreated group. Their findings suggest that AFT may be a more relevant predictor of ethanol consumption than LORR duration alone, and that ethanol pre-exposure alters AFT in a way that increases its strength as a predictor of ethanol consumption [116]. Thus, the effect of prior exposure to alcohol on AFT may be a critical factor in AUD risk in humans. Therefore, AFT may represent a unique endophenotype for AUD, and one goal for future research is to further determine the nature of the contribution of AFT to risk for AUD by examining AFT in subjects across their alcohol-consumption trajectories. These types of longitudinal studies are extremely difficult in humans, but rodent studies are uniquely suited to elucidate the genetic and environmental factors underlying this relationship.

Using a combination of systems and reverse genetic approaches, Blednov et al. [211] sought to investigate the role of *Scn4b* in ethanol-mediated behaviors, including LORR. In humans, the shortest variant of *SCN4B* is part of a co-regulated network that is associated with lifetime alcohol consumption, even though it was not differentially expressed between human alcoholic and control brains [212]. In rodents, transcriptome studies implicated *Scn4b* as a potential candidate gene based on expression levels in the brains of strains with different genetic predispositions for ethanol consumption. However, the direction of the fold-changes in *Scn4b* expression was inconsistent—it varied depending on brain region, strain, and treatment [213,214]. A bioinformatic analysis across multiple datasets also indicated that expression of *Scn4b* was associated with a variety of ethanol-related traits (ethanol consumption, hypothermia, ataxia) in the BXD RI panel [211]. Therefore, in order to functionally determine the contribution of *Scn4b*, Blednov et al. [211] tested whether global or targeted knockdown of *Scn4b* in the central nucleus of the amygdala influenced a variety of ethanol-related behaviors. Functional validation did not support the role of *Scn4b* in ethanol consumption, ataxia, or hypothermia. However, both the *Scn4b* KO mice and the mice with targeted knockdown of *Scn4b* in the central nucleus of the amygdala were significantly more sensitive to the sedative-hypnotic effects of ethanol as measured by LORR. Collectively, these studies illustrate the value in integrating transcriptomic information across brain regions and species with systems genetic approaches to identify plausible candidate genes. Furthermore, they highlight the fact that while transcription studies have many advantages, they can only provide a brief snapshot of heterogeneous cell types at a single point in time. As Blednov et al. [211] note, future research must also interrogate the transcriptome across cell types and stages of the addiction cycle. As compared to humans, rodent models can much more easily provide the type of cellular and temporal resolution that can ultimately lead to the identification and validation of druggable targets.

#### 5.2.3. Hypothermia

The intoxicating effects of ethanol can also be assessed by measuring sensitivity to the hypothermic effects of ethanol. Ethanol-induced hypothermia has been demonstrated in all studied laboratory species [215], is known to be genetically mediated in rodents [84,87,94,183], and is common in intoxicated individuals because it increases loss of body heat due to dilation of blood vessels on the surface of the body. In this paradigm, animals are administered a high dose of ethanol, and their change in body temperature over time is recorded. However, data on body temperature is not regularly collected in most human studies (but see [216]), thus diminishing its translational utility. Still, given its reliability and ease of collection, ethanol-induced hypothermia may have value as an endophenotype if it is correlated with SR in humans. To our knowledge, this has yet to be determined and represents a promising avenue for future research in human subjects.

There is evidence in rodents that the effects of ethanol on hypothermia are associated with its motivational/hedonic properties and may influence consumption [81,217,218,219,220]. In support of this hypothesis, it has been shown that ethanol not only induces changes in the hypothalamic regions involved in thermoregulation, but also in brain areas known to be important for reward/aversion, such as the bed nucleus of the stria terminalis and the central nucleus of the amygdala [221]. In addition, rats self-administered greater amounts of ethanol when exposed to high ambient temperatures (which reduced ethanol-induced hypothermia) as compared to rats who consumed ethanol at normal ambient temperatures [219]. However, when researchers manipulated genotype rather than ambient temperature, they reported somewhat conflicting results. Specifically, mice that were selectively bred for insensitivity (HOT mice) to the hypothermic effects of ethanol drank less ethanol than mice selectively bred for sensitivity (COLD mice) to the hypothermic effects of ethanol [81]. Therefore, additional studies exploring the physiological and molecular mechanisms underlying the effects of ethanol on thermoregulation are needed to better elucidate the relationship between ethanol-induced hypothermia and consumption.

There are a number of bioinformatics platforms that can be leveraged to integrate phenotypic, genomic, transcriptomic, and proteomic data across species to improve our understanding of SR endophenotypes such as ethanol-induced hypothermia. For example, Bubier et al. [222] performed a search of over 60,000 gene sets in the GeneWeaver database (http://www.geneweaver.org; [223]) to identify novel genes related to AUD. First, researchers selected gene sets drawn from mouse genetic mapping studies, differential gene expression studies in rats, and genes expressed in the brains of human alcoholics to generate a list of highly connected hub genes that were found in multiple experimentally derived alcohol-relevant gene sets. Next, they compared these results to genes from Online Mendelian Inheritance in Man (OMIM [224]) that had been previously associated with AUD in humans or to Mammalian Phenotype (MP) Ontology term annotations [225] to ethanol-related traits in mice. They determined that a single gene, *Pafah1b1*, was the most highly connected gene across gene sets and had not been previously annotated to alcohol. To further investigate the role of this gene, they measured a number of ethanol-related traits in heterozygous *Pafah1b1* conditional knockout mice. In the *Pafah1b1* mutant, they observed increased ethanol-induced hypothermia and decreased ethanol preference [222]. Thus, through the use of integrated genomics analyses across rodents and humans, they were able to parse thousands of individual genes to identify and functionally validate a single gene that had not been previously associated with alcohol-related behaviors. Given the vast amount of phenotypic and functional genomic data that exists in rodents, bioinformatics tools such as GeneWeaver not only allow researchers to quickly prioritize genes for functional validation studies in rodents, but can also be used as a priori evidence in human AUD GWAS studies to reduce the burden of multiple testing and lower stringent significance thresholds.

### 5.3. Ethanol-Induced Conditioned Taste Aversion (CTA)

Finally, the subjective effects of alcohol can be assessed in rodents by measuring their sensitivity to its aversive motivational effects. The CTA paradigm is commonly used to assess the unpleasant effects of ethanol. In this procedure, consumption of a flavored (usually sweet) solution is paired with an injection of ethanol. The degree to which the flavored solution is then avoided is interpreted as evidence of the aversive actions of ethanol. CTA is distinct from devaluation procedures, which are used to assess reward disengagement that results from the revision of reward value information and inhibition of previously learned responses [226,227]. Numerous rodent studies have consistently demonstrated large genetic differences in sensitivity to CTA [228,229,230,231], but it is important to note that a high enough dose of ethanol paired with the flavored solution will result in CTA regardless of genotype. Although the neural circuitry underlying ethanol CTA has not been fully elucidated, a number of studies has shown that the mesolimbic dopamine system processes aversive information and governs behavioral responses to aversive stimuli [232,233,234]. There is also evidence suggesting that the lateral habenula may specifically contribute to the aversive effects of ethanol by mediating learning and conditioning processes [235].

In addition, there is a consistently observed inverse relationship between sensitivity to the aversive effects of ethanol and ethanol consumption in both mice and rats [228,229,236,237,238,239,240,241,242,243,244]. For example, in a CTA study comparing alcohol-preferring (P) and alcohol-nonpreferring (NP) rats, it was shown that P rats displayed diminished sensitivity to the aversive effects of ethanol as compared to NP rats [237]. Similarly, high alcohol drinking UChB rats exhibit only modest aversion to ethanol as compared to their low alcohol drinking UChA counterparts [243]. Lastly, both replicates of high alcohol preferring (HAP-1 and HAP-2) and high drinking in the dark (HDID-1 and HDID-2) mice show blunted CTA responses [236,238,239]. Together, these rodent data provide strong evidence that increased sensitivity to the aversive effects of ethanol acts as a protective influence against increased ethanol consumption. There is some support for a similar relationship in humans, where acute responses to the aversive effects of alcohol include facial flushing, nausea, discomfort, and tachycardia. It has been well documented that individuals with increased sensitivity to the flushing response (primarily those of East Asian descent) have a decreased likelihood of developing an AUD [245,246]. However, outside of the context of the flushing response in Asians, the relationship between sensitivity to other aversive effects of alcohol and risk for AUD has not been systematically explored [49]. Given the strength of rodent evidence, this represents a critical avenue for future translational research.

In particular, rodent studies can help determine the ways in which neurodevelopmental changes that occur during adolescence underlie differences in alcohol sensitivity and consumption across the lifespan and influence risk for developing an AUD. Because it is still developing, the adolescent brain is believed to be uniquely vulnerable to alcohol [247,248]. This is of great interest to AUD researchers, given the large body of evidence showing that individuals who initiate drinking by 14 years of age are at increased risk of developing an AUD as compared to those who begin drinking after the age of 19 [249,250,251]. Furthermore, quickly progressing from first drink to first intoxication episode strongly predicts problematic drinking behaviors in adolescents [252,253,254,255]. Thus, initiation of heavy drinking during vulnerable periods may be mediated by the decreased sensitivity of adolescents to the aversive effects of alcohol and contribute to AUD risk. However, there is also evidence that both age of initiation and later AUD share common genetic origin, which implies that interventions aimed at preventing the development of AUD by delaying age at first drink are unlikely to be successful [256]. Resolving these discrepant findings is essential to the development of evidence-based prevention, intervention, and treatment strategies. However, due to ethical restrictions, research on adolescent alcohol sensitivity in humans is understandably limited. Using ethanol CTA, rodent studies have reliably demonstrated that adolescents are less sensitive to the aversive effects of ethanol as compared to adults [257,258,259,260,261,262,263]. In addition, animals exposed to ethanol as adolescents become relatively insensitive to the aversive effects of ethanol as adults [264,265,266,267,268]. Together, these findings suggest that exposure to ethanol during adolescence induces long-lasting decreases in sensitivity to the adverse effects of ethanol and may enable increased ethanol consumption, thus putting individuals at risk for the development of an AUD. Moreover, there is evidence that age-related differences in sensitivity to the aversive effects of ethanol are modulated by genotype. Moore et al. [269] measured CTA during both adolescence and adulthood in eight inbred mouse strains. They found that within any given inbred strain, adolescent mice were less sensitive to the aversive effects of ethanol as compared to adult animals. However, the dose of ethanol required to produce CTA varied as a function of both age and strain [269]. Thus, rodent studies have been able to show that adolescence is characterized in part by an attenuated aversion phenotype, and this phenotype is genetically influenced. Still, additional research is needed to determine the neural underpinnings of blunted sensitivity to the aversive effects of ethanol that is observed in adolescents; and to examine the combined effects of genetic, developmental, and environmental factors that contribute to risk for AUD. Given that most of this research is simply not ethically feasible to perform in adolescent human subjects, we must rely on translational rodent studies to inform our understanding of the neurogenetic variants that influence alcohol aversion during adolescence in order to develop age-specific interventions.

## 6. Conclusions and Future Directions

In this review, we have summarized current findings related to the genetics of alcohol sensitivity in rodents and identified opportunities for bridging human and rodent studies. We remain confident that results gleaned from rodents can continue to be used to (1) prioritize GWAS hits for further investigation, (2) provide further insight into molecular, physiological, and behavioral mechanisms related to alcohol dependence, and (3) identify alternative therapeutic targets that may not be directly nominated by human GWAS. We have highlighted three areas for future translational research that we believe would advance our knowledge about the genetic influences on alcohol sensitivity and subsequently on AUD. First, rodent models of alcohol sensitivity, their genetic determinants, and the influence of previous alcohol exposure should be examined across the BEC curve, at multiple developmental timepoints, and throughout the entire disease progression from initial response to self-administration, tolerance, withdrawal, and relapse. Second, additional research is needed using high-throughput, self-report phenotype measures of alcohol sensitivity in humans in sufficiently large population-based cohorts. This will enable a better understanding of the relationship between alcohol sensitivity, alcohol consumption, and AUD in humans, which in turn can help identify the most biologically and clinically relevant endophenotypes in rodents. Finally, the use of powerful new statistical tools (such as genomic structural equation modeling) and bioinformatics databases (such as GeneNetwork and GeneWeaver) enables the integration of results across endophenotypes, expression data, and species in order to identify gene networks and biological pathways associated with AUD.
